# Supplementary material for: A Comprehensive Assessment of Ultraviolet-Radiation-Induced Mutations in Flammulina filiformis Using Whole-Genome Resequencing
Source: J Fungi (Basel). 2024 Mar 20;10(3):228. doi: 10.3390/jof10030228 (PMC10971301; doi:10.3390/jof10030228)
Supplement: Supplementary file 1 [file jof-10-00228-s001.zip › Supplementary Material S8/KEGG annotation/out/64381550635650.os/KO/out_map/map04745.html]

KEGG PATHWAY: Phototransduction - fly - Reference pathway


|  |  |
| --- | --- |
| **Phototransduction - fly - Reference pathway** |  |

[
Pathway menu
| Organism menu
| Pathway entry
| Show description
| User data mapping
]

Phototransduction is the process that converts the signal of light (photons) into a change of membrane potential in photoreceptor cells. Drosophila visual signaling is initiated with the activation of rhodopsin by light. Rhodopsin is composed of a protein, opsin, covalently linked to a chromophore, 3-hydroxy-11-cis-retinal. Upon absorption of a light photon the chromophore is isomerized from the 11-cis to the all-trans configuration which induces a structural change that activates the opsin. This photoconversion activates heterotrimeric Gq protein via GTP-GDP exchange, releasing the G(alpha)q subunit. G(alpha)q activates PLC, generating InsP3 and DAG from PIP2. DAG may further release polyunsaturated fatty acids (PUFAs) via action of DAG lipase. This reaction leads to the opening of cation-selective channels (trp and trpl genes) and causes the depolarization of the photoreceptor cells. The transduction proteins rhodopsin, G(alpha)q, PLC, PKC, TRP, and TRPL are coordinated by a polymer of INAD scaffold proteins.

|  |  |
| --- | --- |
| Reference pathway | 100% |
